# Nucleus accumbens core acetylcholine receptors modulate the balance of flexible and inflexible cue-directed motivation

**DOI:** 10.1038/s41598-023-40439-4

**Published:** 2023-08-17

**Authors:** Erica S. Townsend, Kenneth A. Amaya, Elizabeth B. Smedley, Kyle S. Smith

**Affiliations:** 1 Department of Psychological and Brain Sciences, Dartmouth College, 3 Maynard Street, Hanover, NH 03755 USA; 2 Department of Neuroscience, Tufts University School of Medicine, Boston, MA USA; 3 Department of Psychology, Temple University, Philadelphia, PA USA

**Keywords:** Motivation, Neuroscience, Reward

## Abstract

Sign-tracking is a conditioned response where animals interact with reward-predictive cues due to the cues having motivational value, or incentive salience. The nucleus accumbens core (NAc) has been implicated in mediating the sign-tracking response. Additionally, acetylcholine (ACh) transmission throughout the striatum has been attributed to both incentive motivation and behavioral flexibility. Here, we demonstrate a role for NAc ACh receptors in the flexibility of sign-tracking. Sign-tracking animals were exposed to an omission contingency, in which vigorous sign-tracking was punished by reward omission. Animals rapidly adjusted their behavior, but they maintained sign-tracking in a less vigorous manner that did not cancel reward. Within this context of sign-tracking being persistent yet flexible in structure, blockade of NAc nicotinic receptors (nAChRs) led to a persistence in the initial sign-tracking response during omission followed by a period of change in the makeup of sign-tracking, whereas blockade of muscarinic receptors (mAChRs) oppositely enhanced the omission-related development of the new sign-tracking behaviors. Later, once omission learning had occurred, nAChR blockade uniquely led to reduced sign-tracking and elevated reward-directed behaviors instead. These results indicate that NAc ACh receptors have opposing roles in maintaining learned patterns of sign-tracking, with nAChRs having a special involvement in regulating the structure of the sign-tracking response.

## Introduction

Animals can attribute motivational value, or incentive salience, to conditioned stimuli (CS) that predict rewards^1^. This CS attraction can manifest as engagement with the cue as if it were the reward itself, also known as sign-tracking^2–4^. Although being drawn into CSs carries an adaptive benefit, cue-reward relationships can change in dynamic environments. Failure to adjust this behavior when those relationships change can relate to suboptimal energy expenditure and even addiction-like CS reactivity^5–7^.

Sign-tracking behavior can be sensitive to reward value^8–11^ and CS-reward contingencies^12^. However, even when animals do show an ability to change sign-tracking behaviors when conditions change, the underlying CS attraction shows a great deal of persistence^12–16^. Omission schedules, also called negative automaintenance^12–15,17^, provide a clear window into this phenomenon. Animals will engage vigorously with a CS that is a lever (e.g., CS biting, grabbing) as part of their normal sign-tracking response. However, when such vigorous cue engagement (specifically, lever deflection) results in reward cancellation (what is called “omission”), animals will learn to reduce doing it. Yet, when one looks closely, the animals do not actually cease sign-tracking. Instead, they change their behaviors to be less vigorous (e.g., CS orienting, sniffing)^12–15^. In this situation, the CS’s incentive salience is preserved as evidenced by their continued motivational persistence towards the CS, yet animals can flexibility adjust how they engage with the CS behaviorally. Omission schedules thus provide an opportunity to study brain mechanisms for how reward cues compel an enduring attraction from animals while allowing flexibility in its behavioral expression.

The nucleus accumbens core (NAc) is involved in motivation and sign-tracking^18–23^. This region hosts a small population of tonically active cholinergic interneurons (ChIs) that are the primary source of acetylcholine (ACh)^24^. ACh receptors consist of two major subtypes: ionotropic nicotinic receptors (nAChRs^25^) and G-protein coupled muscarinic receptors (mAChRs^26^). nAChRs are primarily expressed on dopaminergic terminals from the ventral tegmental area (VTA^27,28^), while mAChRs are primarily autoreceptors located on ChIs themselves^29^. ACh receptors can modulate dopamine (DA) signaling^27,29–33^, including cue-evoked DA release that is required for the maintenance and acquisition of the sign-tracking response^23^. Striatal ACh transmission is known to be involved in behavioral flexibility and motivation^30,34–41^. Prior studies have further shown opposing effects in the modulation of motivated behaviors resulting from the blockade of individual ACh receptor subtypes^39^, likely in part because of their opposing actions on striatal activity in which mAChR autoreceptor binding could reduce ACh signaling while nAChR postsynaptic binding could increase ACh signaling.

It is plausible that NAc ACh transmission plays an important role in regulating how sign-tracking behaviors are expressed, particularly under situations like omission that result in a maintenance of cue attraction but an adjustment of its expression in behavior. Here, we tested the hypothesis that blockade of nAChRs or mAChRs in sign-tracking animals would affect the ability of animals to change their expression of sign-tracking behaviors when exposed to omission learning.

## Methods

### Experiment 1: nicotinic receptor blockade during introduction of an omission schedule

#### Subjects

Eighteen PN 70–90 sign-tracking male (n = 10) and female (n = 8) Long Evans rats (Charles River, Indianapolis, IN) were single housed, and on a 12 h light/dark cycle (lights on at 7 AM). Experiments were conducted during the light cycle. Rats were food restricted (7–15 g of standard chow per day) to 85% of their free-feeding weight throughout testing. Water was available ad libitum. This stock of rat nearly unanimously develops sign-tracking behaviors, which suits our goals of analyzing sign-tracking behavior. All procedures involving animals in this study were executed with the approval of the Dartmouth College Institutional Care and Use Committee and all methods are in accordance with both AAALAC International guidelines and ARRIVE guidelines.

#### Surgical procedures

Under aseptic conditions, rats were anesthetized with isoflurane gas and placed in a stereotaxic apparatus (Stoelting, Kiel, WI). 22-gauge, stainless steel guide cannulas (P1 Technologies, Roanoke, VA) were implanted bilaterally, 1 mm above the NAc infusion site (AP + 1.3 mm, ML ± 1.8, DV − 6.2 relative to Bregma; coordinates were chosen based on a prior NAc drug infusion study^39^). Following surgery, rats were given intraperitoneal (IP) injections of 3 mg/kg of ketoprofen, 3 mL of 0.9% sterile saline, and 0.02 mL of enrofloxacin for 3 days. Animals recovered for 5 days with ad libitum food and water. Food restriction resumed a minimum of 5 days before behavioral procedures resumed.

#### Testing apparatus

Tests were conducted in identical chambers (20 × 30.5 × 29 cm; Med Associates, St. Albans, VT) enclosed in sound- and light-attenuating cabinets (62 × 56 × 56 cm) equipped with a fan for airflow and background noise (~ 68 dB) and illuminated by a house light on the back wall. Chambers contained two retractable levers on either side of a recessed magazine in which food rewards were delivered. Lever deflections and magazine entries were recorded using the MED-PC IV software. Videos were recorded for behavioral analysis.

#### Sign-tracking training

Training began with a 30-min magazine acclimation session where one pellet was delivered approximately every 30 s. Rats then received 12 days of Pavlovian sign-tracking (ST) training sessions. The first 10 ST sessions of were given over 10 consecutive days, and, following surgery and post-operative procedures, the rats received 2 more training sessions for reacquisition. ST training sessions contained 25 CS+ trials in which a 10-s presentation of a retractable lever was followed by noncontingent 45 mg grain pellet (BioServ, Frenchtown, NJ) delivery, and 25 CS− trials in which the 10-s presentation of the other retractable lever was followed by nothing. CS+ and CS− levers were counterbalanced across animals. Trials were pseudorandomized so that no more than two of the same trial type were followed in sequence. Intertrial intervals were variable (ranging from 45 to 75 s) with an average length of 1 min. Sessions lasted approximately 1 h.

#### Omission testing

After completing 12 training days, rats underwent 7 days of omission testing. Like the Pavlovian training schedule, these sessions contained 25, 10-s CS+ trials and 25, 10-s CS− trials. Under the omission condition, a deflection of the CS+ lever presentation would cancel reward delivery for that trial. CS+ trials in which the rats did not deflect the lever were rewarded. This schedule of omission is also called negative automaintenance in the literature.

#### Drug and infusion procedures

Animals were handled for one week before training and in the days leading up to infusion procedures. Before each of the first 5 omission testing sessions, rats were bilaterally infused with either mecamylamine (10 μg/side; Tocris Bioscience, Bristol, UK), a nonselective nicotinic receptor antagonist, or sterile artificial cerebrospinal fluid (ACSF; Tocris Bioscience). Rats were gently held while either the drug or ACSF were infused into the NAc at a volume of 0.5 μL over 1 min through injectors (P1 Technologies, Roanoke, VA) inserted into the guide cannulas, protruding 1 mm ventral of the cannula tips. The injectors rested in the cannulas for 1 min after infusion, and rats were kept in a holding chamber for 10 min before beginning the task.

#### Histological procedures

Following experiments, rats were anesthetized with sodium pentobarbitol (100 mg/kg) and perfused intracardially with 0.9% saline, followed by 10% formalin. Brains were removed and stored in 20% sucrose for 24 h, then sectioned at 60 μm. Sections were mounted on slides and cover slipped with a DAPI-containing mounting medium (Vectashield; Vector Laboratories, Burlingame, CA) to verify cannula placements. Placement maps were created by manually transcribing the most ventral affected regions onto printed images, and then transcribed digitally via Adobe Illustrator (26.0.1 Adobe Creative Cloud).

#### Data analysis

Lever press rates were calculated as presses per minute (PPM) by dividing the total presses for a lever by the total minutes of lever availability. Statistical tests were carried out using R (version 4.2.2)^42^. Individual linear mixed models (LMMs) were analyzed with “lme4” from CRAN^43^ to analyze the effects of dependent variable responding (e.g., lever PPM, magazine entries). Models were created for the two major phases in the experiment: sign-tracking training (sessions 1–12), and baseline (an average of the final 3 sign-tracking training sessions) through omission testing (baseline + sessions 13–19). This baseline calculation was used to account for any variation between groups during training to better compare PPM and magazine entries between groups during omission. In addition, PPM and magazine entries during omission testing were normalized to this baseline. Reported statistics include parameter estimates (β values), 95% confidence intervals (CI), and *p*-values (R; “lmerTest”)^44^. LMMs were used because they consider aspects of the data structure that repeated measures ANOVA cannot and allows for safer generalization to larger populations. We present magazine entry data differently than PPM lever interactions because we do not compare or combine these data, and because they constitute two rather different types of behaviors. Moreover, we find that magazine entry lengths can be variable (e.g., an animal may enter the magazine and remain inside of it for several seconds, but another may poke their head in for a very short period of time), leading to the chosen magazine reporting method to be most reliable.

Videos were hand-scored for 4 sessions: session 12, 13, 17, and 19. Within each session for a given animal, six 10-s CS+ trials were scored: the 1st, 5th, 10th, 15th, 20th, and 25th trials. Behaviors that occurred on the odd seconds of the trial were recorded for a total of 30 behaviors scored per session, per animal. Behaviors were scored into 7 categories: lever bites, lever grabs (one paw on each side of the lever), lever contacts (one-pawed touch on any side of the lever), lever sniffs (snout close to or touching the lever), lever orients (staring at the lever), magazine-directed behaviors (magazine entry or orienting), and other non-CS+ directed behaviors (orienting or approaching CS− lever wall, orienting away from CS+ lever, ignoring CS+ lever). LMMs were analyzed similarly to lever PPM and magazine entry models, with each behavior as the dependent variable. All plots were created using the “ggplot2” package for R^45^ and formatted in Adobe Illustrator.

### Experiment 2: muscarinic receptor blockade during introduction of an omission schedule

Subjects were 18 experimentally naïve sign-tracking male (n = 11) and female (n = 7) Long Evans rats. This experiment was run identically to Experiment 1 except for the drug. After completing 12 days of training, rats underwent 7 days of omission testing. Ten minutes prior to the first 5 omission sessions, rats were bilaterally infused with either scopolamine (10 μg/side; Tocris Bioscience, Bristol, UK), a nonselective muscarinic receptor antagonist, or an equivalent volume of sterile ACSF (Tocris Bioscience).

### Experiment 3: nicotinic receptor blockade during sign-tracking overtraining

Subjects were 18 experimentally naïve sign-tracking male (n = 9) and female (n = 9) Long Evans rats. This experiment was run identically to Experiment 1, except rather than omission, the rats received additional overtraining days. Thus, after 12 days of sign-tracking training, rats underwent 7 days of overtraining where they continued the sign-tracking training. Infusion procedures for mecamylamine proceeded as described in Experiments 1 for the first 5 of 7 days of overtraining.

## Results

### Experiment 1: nicotinic receptor blockade during introduction of an omission schedule

#### Sign-tracking presses per minute

**Figure 1:**
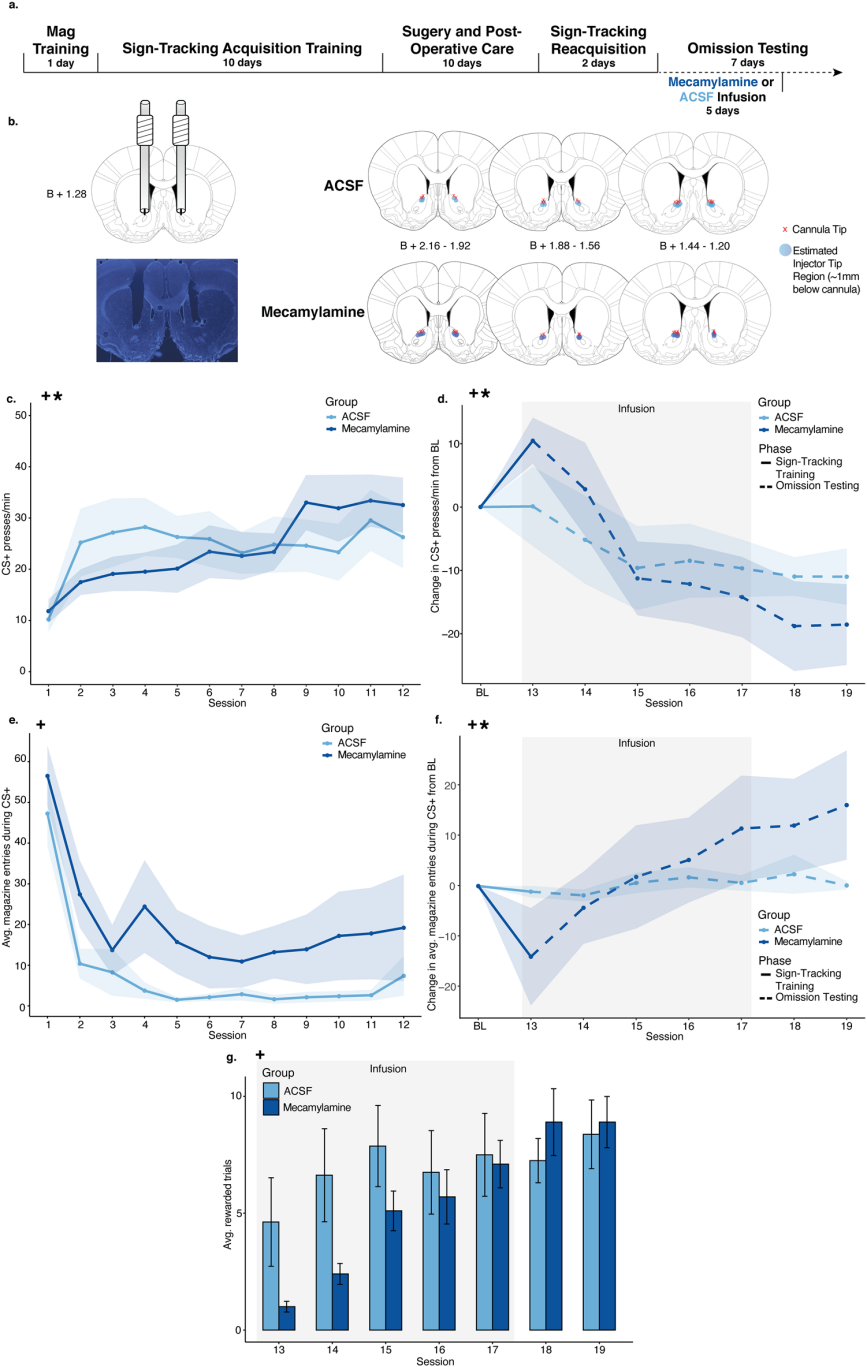
Experiment 1 results. (**A**) Timeline of behavioral training, surgical procedures, and infusions. (**B**) Cannula implant histology. All red x’s mapped indicate the position of the bottom of the cannula and points indicate the estimated position of the needle, in the A/P coordinate closest to the center of the cannula implantation site. (**C**) Presses per minute (PPM) on the CS+ lever over the 12 sign-tracking training sessions for the mecamylamine (dark blue) and ACSF (light blue) groups. Shaded sessions indicate infusion sessions. (**D**) Change in presses per minute (PPM) on the CS+ lever from a 3-session baseline created for each animal by averaging PPM on the last 3 sessions of sign-tracking training. All 7 omission sessions were normalized to the 3-session baseline for the mecamylamine (dark blue) and ACSF (light blue) groups. Shaded sessions indicate infusion sessions. (**E**) Average magazine entries during 10-s CS+ presentations over the 12 sign-tracking training sessions for the mecamylamine (dark blue) and ACSF (light blue) groups. Shaded sessions indicate infusion sessions. (**F**) Change in average magazine entries during the CS+ lever presentations from a 3-session baseline created for each animal by averaging PPM on the last 3 sessions of sign-tracking. All 7 omission sessions were normalized to the 3-session baseline for the mecamylamine (dark blue) and ACSF (light blue) groups. Shaded sessions indicate infusion sessions. (**G**) Average rewarded trials over the 7 omission sessions for the mecamylamine (dark blue) and ACSF (light blue) groups. Shaded sessions indicate infusion sessions. For all graphs, points or bars show mean of each session and error ribbons or bars show ± SEM. Plus symbols (+) indicate significant main effects of session, and asterisks (*) indicate significant interactions between session and group.

To compare group CS+ PPM with respect to time during sign-tracking acquisition training and omission testing, LMMs used PPM as the dependent variable by fixed effects of session, group, and the interaction between session and group. During sign-tracking acquisition training (Fig. 1C), there was no main effect of group (est: − 0.27; CI: − 5.44 to 4.90; p = 0.919), but a main effect of session (est: 4.24; CI: 2.22–6.25; **p < 0.001**) and an interaction between group and session (est: 2.26; CI: 0.24–4.28; **p = 0.028**) were found. It was unclear why groups might diverge prior to any manipulation. However, to normalize for this interaction, we calculated a baseline for behavioral comparisons by averaging animals’ PPM in the final 3 sign-tracking acquisition sessions and included this in the LMM comparing CS+ PPM from baseline through omission testing (Fig. 1D). Thus, effects of omission and/or ACh manipulations for each rat were relative to their own baseline sign-tracking behaviors. In this model, no main effect of group was found (est: − 0.43; CI: − 6.95 to 6.09; p = 0.896), but a main effect of session (est: − 6.47; CI: − 8.53 to − 4.41; **p < 0.001**) and a significant interaction (est: − 2.51; CI: − 4.57 to − 0.45; **p = 0.017**) were found, suggesting differences in the rate of CS+ PPM between groups as animals underwent omission testing. CS− PPM were also analyzed with no significant effect of group or an interaction between group or session during training or omission (see Supplementary Fig. 1A; Supplementary Table 1).

#### Magazine entries during and after CS+ lever presentations

To compare mean group magazine entries with respect to time during the two major phases of the experiment, LMMs used the total magazine entries during only CS+ presentations as the dependent variable by fixed effects of session, group, and the interaction between session and group, with random intercepts for individual animal start points included. During sign-tracking training (Fig. 1E), the model indicated a main effect of session (est: − 6.91 entries; CI: − 10.01 to − 3.81; **p = < 0.001**), but not group (est: 5.79 entries; CI: − 1.99 to 13.57; p = 0.144) or group and session interactions (est: − 0.60 entries; CI: − 3.70 to 2.50; p = 0.705). All animals decreased magazine entries during cue presentations across training, and both groups did so similarly. A similar 3-session average baseline was created, and omission PPM was normalized to this baseline and included in a LMM to compare group magazine entries during omission (Fig. 1F). This model revealed significant main effects of session (est: 4.42; CI: 1.17–7.68; **p = 0.008**) and a significant interaction between session and group (est: 3.68; CI: 0.43–6.94; **p = 0.027**), but no main effect of group (est: 1.62; CI: 6.43–9.67; p = 0.691). This reflected the result that magazine entries of animals in the mecamylamine group increased at a much steeper rate than animals in the ACSF group during omission.

Per-group magazine entries during the 10 s post-CS+ presentation period (i.e., reward delivery period) were compared using similar LMMs with the total magazine entries during this post-CS+ period as the dependent variable (see Supplementary Fig. 2A,B; Supplementary Table 2). During sign-tracking acquisition, there were no significant effects of group or an interaction between session and group. During omission testing, a significant main effect of group was found (see Supplementary Table 2), in which animals that received mecamylamine remain relatively stable in their post-CS+ presentation magazine entries from sign-tracking acquisition through omission testing, while animals in the ACSF group may have driven this main effect as they decreased their post-CS+ entries significantly. However, this finding is explainable by the mecamylamine animals having a lower average baseline magazine entry level leading into omission and control animals trending towards that same level as omission days played out (compare Supplementary Fig. 2A,B); thus, the statistical difference during omission in the baseline-normalized data does not reflect a meaningful difference between the groups related to omission learning.

#### Rewarded trials during omission

To compare the average rewarded trials during omission testing with respect to session, LMMs used the average rewarded trials per session as the dependent variable by fixed effects of session, group, and the interaction between session and group, with random intercepts for individual animal start points included. During the 7 sessions of omission testing (Fig. 1G), a significant main effect of session (est: 1.82; CI: 0.81–2.83; **p < 0.001**) was found, but no significant main effect of group (est: − 0.71; CI: − 2.98 to 1.56; p = 0.540). A near-significant interaction between group and session was observed (est: 0.95; CI: − 0.06 to 1.96; p = 0.065), indicating that animals who received mecamylamine were trending towards lower reward retrieval in contrast to their ACSF counterparts upon the first several sessions of omission testing. This trend naturally mirrors the lever pressing data, such that greater pressing behavior generally led to fewer rewards.

### Experiment 2: muscarinic receptor blockade during introduction of an omission schedule

#### Sign-tracking presses per minute

**Figure 2:**
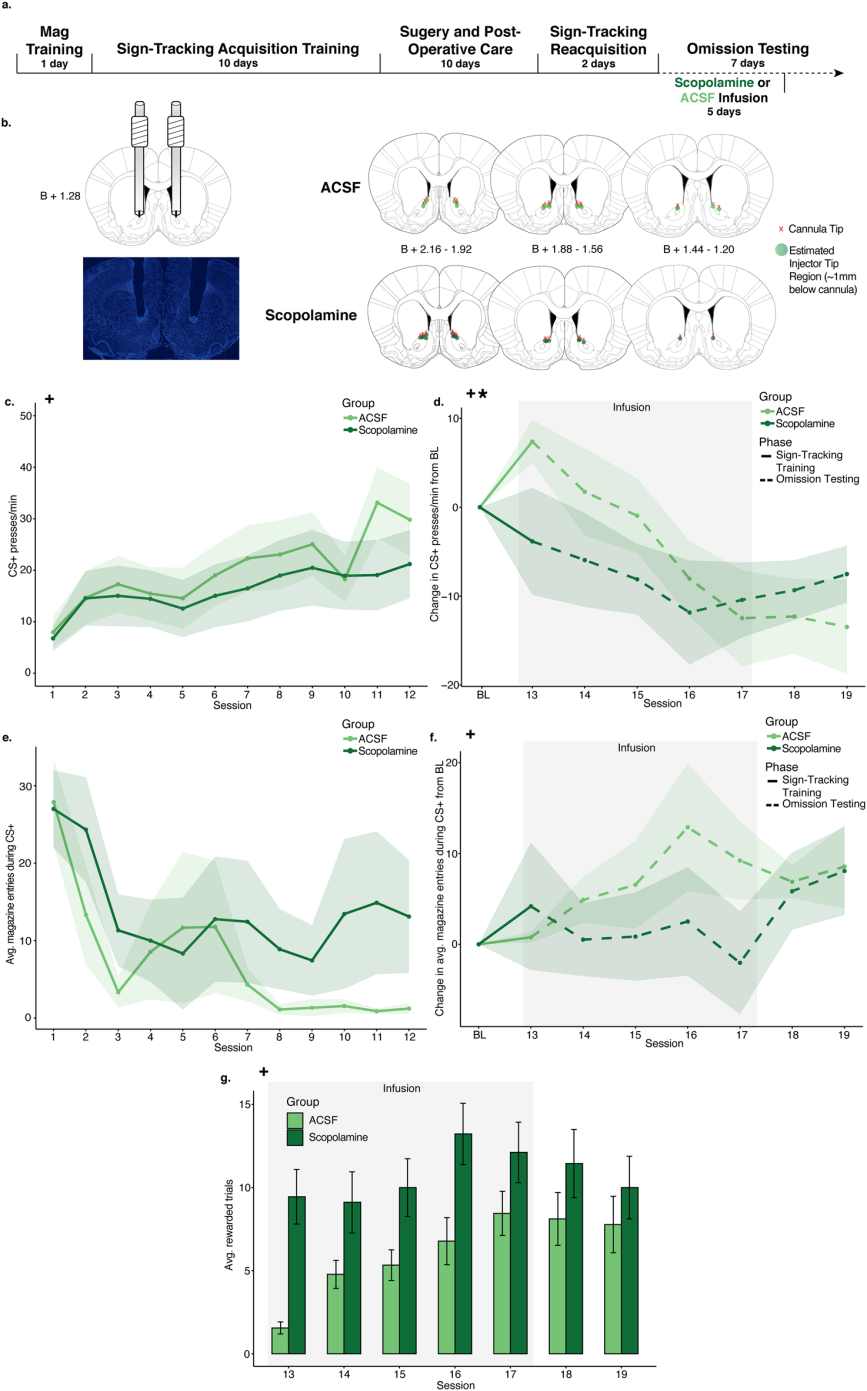
Experiment 2 results. (**A**) Timeline of behavioral training, surgical procedures, and infusions. (**B**) Cannula implant histology. All red x’s mapped indicate the position of the bottom of the cannula and points indicate the estimated position of the needle, in the A/P coordinate closest to the center of the cannula implantation site. (**C**) Presses per minute (PPM) on the CS+ lever over the 12 sign-tracking training sessions for the scopolamine (dark green) and ACSF (light green) groups. Shaded sessions indicate infusion sessions. (**D**) Change in presses per minute (PPM) on the CS+ lever from a 3-session baseline created for each animal by averaging PPM on the last 3 sessions of sign-tracking training. All 7 omission sessions were normalized to the 3-session baseline for the scopolamine (dark green) and ACSF (light green) groups. Shaded sessions indicate infusion sessions. (**E**) Average magazine entries during 10-s CS+ presentations over the 12 sign-tracking training sessions for the scopolamine (dark green) and ACSF (light green) groups. Shaded sessions indicate infusion sessions. (**F**) Change in average magazine entries during the CS+ lever presentations from a 3-session baseline created for each animal by averaging PPM on the last 3 sessions of sign-tracking. All 7 omission sessions were normalized to the 3-session baseline for the scopolamine (dark green) and ACSF (light green) groups. (**G**) Average rewarded trials over the 7 omission sessions for the scopolamine (dark green) and ACSF (light green) groups. Shaded sessions indicate infusion sessions. For all graphs, points or bars show mean of each session and error ribbons or bars show ± SEM. Plus symbols (+) indicate significant main effects of session, and asterisks (*) indicate significant interactions between session and group.

Group CS+ PPM was analyzed with respect to session during sign-tracking acquisition training and omission testing using LMMs. These models used lever PPM as the dependent variable by fixed effects of session, group, and the interaction between session and group. During the 12 sessions of sign-tracking training (Fig. 2C), there was a significant effect of session (est: 4.64; CI: 1.94–7.35; **p = 0.001**), but no effect of group (est: − 1.96; CI: − 8.77 to 4.86; p = 0.572) or session/group interaction (est: − 1.29; CI: − 3.99 to 1.42; p = 0.350), indicating that both groups acquired the sign-tracking response similarly prior to manipulations. As in Experiment 1, a baseline was created by averaging the last 3 days of sign-tracking training. Omission PPM were normalized to this baseline, and included in a LMM comparing group CS+ PPM through omission testing (Fig. 2D). In this model, a significant main effect of session (est: − 4.65; CI: − 6.56 to − 2.74; **p < 0.001**) and a significant interaction between session and group (est: 1.99; CI: 0.08–3.90; **p = 0.042**) were found, suggesting a difference in the rate at which animals acquired the omission task. No significant main effect of group was found (est: − 1.18; CI: − 5.73 to 3.37; p = 0.609). Thus, animals that received scopolamine altered sign-tracking responses more quickly than controls during omission. CS− PPM were also analyzed with no significant effect of group or an interaction between group or session during training or omission (see Supplementary Fig. 1B; Supplementary Table 3).

#### Magazine entries during and after CS+ lever presentations

To compare magazine entries between groups with respect to time during the three major phases of the experiment, linear mixed models used the total magazine entries during only CS+ presentations as the dependent variable by fixed effects of session, group, and the interaction between session and group, with random intercepts for individual animal start points included. During the first 12 days of sign-tracking acquisition training (Fig. 2E), the models indicated no significant effects of session (est: − 0.54 entries; CI: − 5.69 to 4.61; p = 0.837), group (est: − 0.87 entries; CI: − 7.70 to 5.96, p = 0.803), or significant interactions between session and group (est: − 1.63 entries; CI: − 6.78 to 3.52; p = 0.534). Overall, no changes in magazine entries during cue presentations were prevalent. A 3-session average baseline was created, magazine entries were normalized to this baseline, and then subsequentially included in a LMM to compare group magazine entries during omission (Fig. 2F), which revealed a significant effect of session (est: 2.31; CI: 0.42–4.19; **p = 0.017**), but no significant effect of group (est: − 1.86; CI: − 5.80 to 2.07; p = 0.351) or an interaction between session and group (est: − 0.70; CI: − 2.58 to 1.19; p = 0.466). These models indicate no differences between groups on magazine entries during cue presentations under omission conditions.

Group magazine entries during the 10 s post-CS+ presentation period (i.e., reward delivery period) were compared using similar LMMs with the total magazine entries during this post-CS+ period as the dependent variable (see Supplementary Fig. 2C,D; Supplementary Table 4). During sign-tracking acquisition and omission testing, there were no significant effects of group or an interaction between session and group, indicating that both groups remained relatively stable in their post-CS+ magazine entries and thus, reward retrieval.

#### Rewarded trials during omission

To compare the average rewarded trials during omission testing with respect to session, LMMs used the average rewarded trials per session as the dependent variable by fixed effects of session, group, and the interaction between session and group, with random intercepts for individual animal start points included. During the 7 sessions of omission testing (Fig. 2G), no main effect of session (est: 1.32; CI: − 0.07 to 2.71; p = 0.063) was found, nor a significant interaction between session and group (est: − 0.72; CI: − 2.11 to 0.68; p = 0.312). A trending main effect of group was observed (est: 2.33; CI: − 0.22 to 4.87; p = 0.073), indicating that animals who received scopolamine were trending towards higher reward receipt, which was in keeping with their lower pressing behavior.

### Experiment 3: nicotinic receptor blockade during sign-tracking overtraining

#### Sign-tracking presses per minute

**Figure 3:**
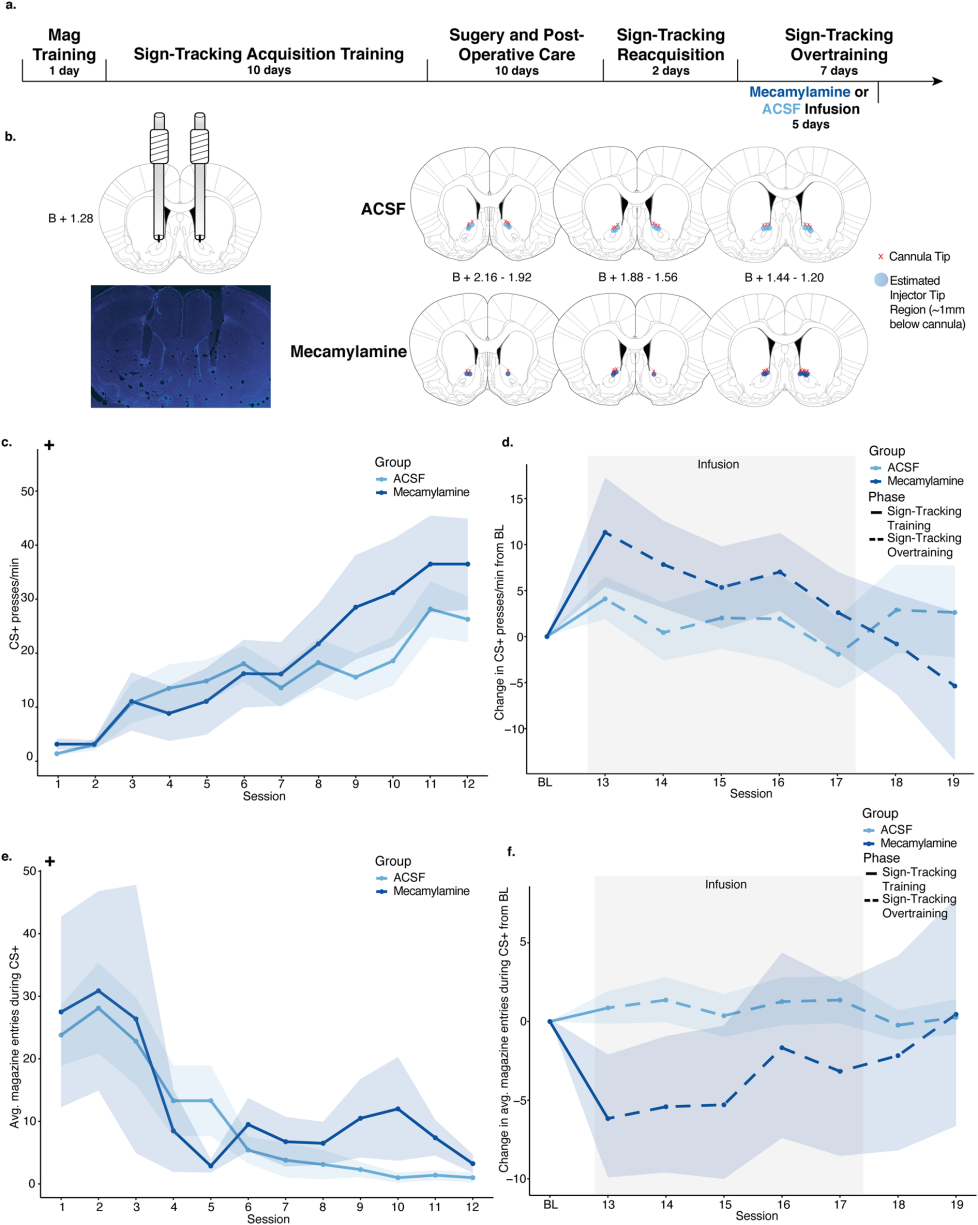
Experiment 3 results. (**A**) Timeline of behavioral training, surgical procedures, and infusions. (**B**) Cannula implant histology. All red x’s mapped indicate the position of the bottom of the cannula and points indicate the estimated position of the needle, in the A/P coordinate closest to the center of the cannula implantation site. (**C**) Presses per minute (PPM) on the CS+ lever over the 12 sign-tracking training sessions for the mecamylamine (dark blue) and ACSF (light blue) groups. Shaded sessions indicate infusion sessions. (**D**) Change in presses per minute (PPM) on the CS+ lever from a 3-session baseline created for each animal by averaging PPM on the last 3 sessions of sign-tracking. All 7 overtraining sessions were normalized to the 3-session baseline for the mecamylamine (dark blue) and ACSF (light blue) groups. Shaded sessions indicate infusion sessions. (E) Average magazine entries during 10-s CS+ presentations over the 12 sign-tracking training sessions for the mecamylamine (dark blue) and ACSF (light blue) groups. Shaded sessions indicate infusion sessions. (F) Change in average magazine entries during the CS+ lever presentations from a 3-session baseline created for each animal by averaging PPM on the last 3 sessions of sign-tracking. All 7 overtraining sessions were normalized to the 3-session baseline for the mecamylamine (dark blue) and ACSF (light blue) groups. Shaded sessions indicate infusion sessions. For all graphs, points show mean of each session and error ribbons show ± SEM. Plus symbols (+) indicate significant main effects of session.

To compare group PPM with respect to session during the three major phases of the experiment, LMMs used PPM as the dependent variable by fixed effects of session, group, and the interaction between session and group. The models indicated that all rats acquired the sign-tracking response similarly during the 12 sessions of training (Fig. 3C), showing a significant main effect of session (est: 9.12; CI: 6.25–11.99; **p < 0.001**), and no main effects of group (est: 1.75; CI: − 3.65 to 7.16; p = 0.523) nor a session/group interaction (est: 2.22; CI: − 0.65 to 5.09; p = 0.128). A series of 7 overtraining sessions followed in which there was no task change (i.e., no omission). A model was created including PPM normalized to the baseline of the final 3 sessions of sign-tracking acquisition training, as in Experiments 1 and 2 (Fig. 3D). This model indicated no significant effects of session (est: − 1.46; CI: − 3.70 to 0.78; p = 0.200), group (est: 0.99; CI: − 3.42 to 5.40, p = 0.658), nor an interaction between session and group (est: − 1.60; CI: − 3.85 to 0.64; p = 0.160). This indicates no significant effects of mecamylamine infusion on sign-tracking responses, and thus no effect on motivation generally, when there was no change to the task rules. CS− PPM were also analyzed with no significant effects of group or an interaction between group or session during training or omission (see Supplementary Fig. 1C; Supplementary Table 5).

#### Magazine entries during CS+ lever presentations

To compare mean group magazine entries with respect to time during the three major phases of the experiment, linear mixed models used the total magazine entries during only CS+ presentations as the dependent variable by fixed effects of session, group, and the interaction between session and group, with random intercepts for individual animal start points included. During sign-tracking training (Fig. 3E), the model indicated a main effect of session (est: − 7.84 entries; CI: − 12.91 to − 2.78; **p = 0.003**), but not group (est: 1.23 entries; CI: − 3.18 to 5.65; p = 0.582) nor group and session interactions (est: 0.67 entries; CI: − 4.40 to 5.74; p = 0.795). This demonstrates that there were no effects of mecamylamine on magazine entries during the CS+ presentations. A 3-session average baseline was used to normalize magazine entries and was included in a LMM to compare group magazine entries during omission (Fig. 3F), which revealed no significant effects of session (est: 0.42; CI: − 1.28 to 2.12; p = 0.624), group (est: − 1.79; CI: − 5.72 to 2.14; p = 0.369), or interaction between session and group (est: 0.50; CI: − 1.20 to 2.20; p = 0.564). These models show no effects of mecamylamine on magazine entries when no task changes occur.

Group magazine entries during the 10 s post-CS+ presentation period (i.e., reward delivery period) were compared using similar LMMs with the total magazine entries during this post-CS+ period as the dependent variable (see Supplementary Fig. 2E,F; Supplementary Table 6). During sign-tracking acquisition and omission testing, there were no significant effects of group or an interaction between session and group, indicating no significant changes in post-CS+ magazine entries, and thus, reward retrieval, due to mecamylamine infusion alone.

### Experiments 1 and 2 response microstructure analysis

**Figure 4:**
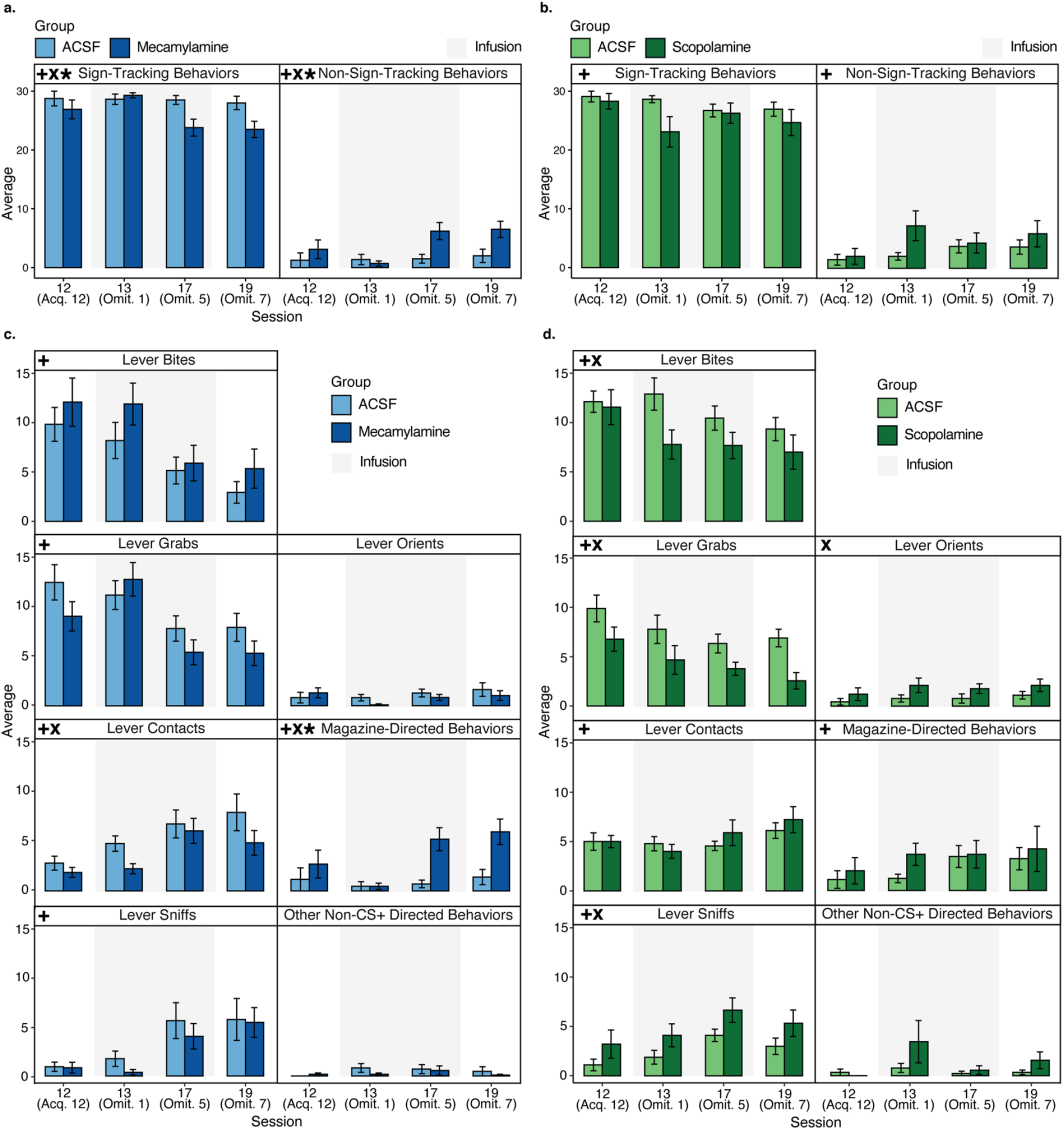
Microstructure of behavior in Experiments 1 and 2. (**A**) Average of individual scored behaviors collapsed into Sign-Tracking Behaviors (lever bites, lever grabs, lever contacts, lever sniffs, lever orients) or Non-Sign-Tracking Behaviors (magazine-directed behaviors, other non-CS+ directed behaviors) during Experiment 1 in the mecamylamine (dark blue) and ACSF (light blue) groups. (**B**) Average of individual scored behaviors collapsed into Sign-Tracking Behaviors (lever bites, lever grabs, lever contacts, lever sniffs, lever orients) or Non-Sign-Tracking Behaviors (magazine-directed behaviors, other non-CS+ directed behaviors) during Experiment 2 in the scopolamine (dark green) and ACSF (light green) groups. (**C**) Average scored behaviors exhibited during 10-s CS+ presentations during Experiment 1 in the mecamylamine (dark blue) and ACSF (light blue) groups. Infusion sessions are indicated by gray shading. (**D**) Average scored behaviors exhibited during 10-s CS+ presentations during Experiment 2 in the scopolamine (dark green) and ACSF (light green) groups. Infusion sessions are indicated by gray shading. For all graphs, bars show the mean and error bars show ± SEM, and asterisks represent significant linear mixed model group and/or interaction effects. Plus symbols (+) indicate significant main effects of session, X symbols (x) indicate significant main effects of group, and asterisks (*) indicate significant interactions between session and group.

Lever presses and magazine entries provide a valuable readout of task behaviors, but they can neglect important microstructural details of behavior that become critical aspects of how animals react to the omission schedule. To compare changes in the microstructure of sign-tracking responses between groups from the last day of sign-tracking training through the end of omission testing, LMMs were employed using the mean of each behavioral response type (Fig. 4) as the dependent variable by fixed effects of session, group and the interaction between session and group.

We first categorized behaviors into those that reflect sign-tracking (CS orient, sniff, contact, bite, grab) and those that do not (magazine directed behavior and non-task behavior) (Fig. 4A,B). In Experiment 1, significant effects of session (est: − 1.02; CI: − 1.71 to − 0.32; **p = 0.005**), group (est: − 1.30; CI: − 2.53 to − 0.06; **p = 0.040**), and an interaction between session and group (est: − 0.75; CI: − 1.44 to − 0.06; **p = 0.035**) were seen in sign-tracking behaviors (Fig. 4A). Significant effects of session (est: 1.02; CI: 0.32–1.71; **p = 0.005**), group (est: 1.30; CI: 0.06–2.53; **p = 0.040**) and an interaction between session and group (est: 0.75; CI: 0.06–1.44; **p = 0.035**) were additionally observed in non-sign-tracking behaviors through omission (Fig. 4A). These models indicate a decrease in, however not complete loss of, sign-tracking behaviors only in animals that received mecamylamine during omission. Non-sign-tracking behaviors were augmented alongside this decrease in sign-tracking behaviors, driven by rising magazine entries during omission.

In Experiment 2, a significant effect of session (est: − 0.89; CI: − 1.67 to − 0.11; **p = 0.026**), but no effect of group (est: − 1.11; CI: − 2.80 to 0.57; p = 0.192) nor an interaction between session and group (est: 0.04; CI: − 0.74 to 0.82; p = 0.924) were seen in sign-tracking behaviors (Fig. 4B). A significant effect of session (est: 0.93; CI: 0.12–1.73; **p = 0.024**), however no effect of group (est: 1.06; CI: − 0.59 to 2.70; p = 0.204) nor an interaction between session and group (est: 0.03; CI: − 0.78 to 0.83; p = 0.951) were observed in non-sign-tracking behaviors through omission (Fig. 4B). These models indicate no alterations to the type of responding (i.e., sign-tracking, or non-sign-tracking) animals engaged in either drug treatment group.

Concerning behavioral details, as can be seen in Fig. 4, omission led in control rats to a reduction in specific sign tracking behaviors that related to lever deflections and reward loss, namely a reduction in CS+ lever bites and grabs. In parallel, there was a rise in behaviors that did not lead to lever deflection, specifically CS+ lever sniffs and light contacts. Thus, animals did not stop sign-tracking during omission, but rather changed the makeup of their sign-tracking response to avoid lever deflections and reward loss. Comparing mecamylamine to these control animals, in Experiment 1 there was a significant reduction in light CS+ lever contacts and a significant increase (in later omission days) of magazine-directed behavior (Fig. 4C). The CS+ lever contact model revealed an effect of group (est: − 0.97; CI: − 1.81 to − 0.13; **p = 0.024**), and the magazine-directed behavior model revealed an effect of group (est: 1.43; CI: 0.36–2.49; **p = 0.010**) and a session/group interaction (est: 0.82; CI: 0.16–1.48; **p = 0.015**). These models along with others (see Supplementary Table 7) indicate a shift towards mid-CS+ presentation magazine “checking” in animals that received mecamylamine, however no changes occur in high contact behaviors such as biting and grabbing (although we do note trends in which mecamylamine animals tend to engage in these behaviors more; see Fig. 4A).

In Experiment 2, significant effects of group were seen in models predicting bites (est: − 1.35; CI: − 2.64 to − 0.05; **p = 0.042**), grabs (est: − 1.64; CI: − 2.86 to − 0.42; **p = 0.009**), orients (est: 0.51; CI: 0.05–0.97; **p = 0.029**), and sniffs (est: 1.15; CI: 0.08–2.22; **p = 0.035**; Fig. 4D). This indicates an increase in behaviors that may result in less deflections (e.g., orients and sniffs) and a decrease in more vigorous behaviors (e.g., bites and grabs) that cause more deflections in animals that received scopolamine. Thus, behavioral alterations extended further than a general decrease in PPM during omission. Models for other behaviors in Experiments 1 and 2 did not show significant effects of group or interactions (see Fig. 4 and Supplementary Tables 7, 8).

## Discussion

Reward-predictive cues have a powerful ability to motivate behavior towards the cues themselves, a behavior known as sign-tracking^1,4^. As sign-tracking responses to cues develop, they can be sensitive to the cue-reward relationship^1,3,9,10,12,46^. Yet, once sign-tracking is established as a response, it can be difficult to stop. This is seen when sign-tracking animals are faced with an omission schedule. In the omission schedule used here, sign-tracking that resulted in deflection of the lever canceled reward delivery. Animals initially continue to engage with the cue in ways that produce deflections even when doing so cancels reward delivery, but then learn to interact with the lever in a way that allows rewards to be delivered by restructuring their cue-directed responses to avoid deflections that omit reward delivery. Reacting to omission in this way therefore involves a mix of motivational flexibility (i.e., changing the response phenotype) and persistence (i.e., continuing responding in some manner). The NAc ACh system shows promise as a potential mechanism of not just motivation to respond to a cue itself, but of maintenance of motivation when cue-reward relationships change. Here, we found that activity at the two major subtypes of ACh receptors have significant and opposing functions in how animals restructure their sign-tracking when navigating an omission procedure. In other words, ACh signaling in the NAc plays a special role in how dynamic animals’ motivational attraction to reward cues is.

Specifically, we tested the hypothesis that blocking NAc ACh activity at nAChRs or mAChRs would specifically affect the flexibility of sign-tracking (i.e., how animals restructure their interaction with reward cues during omission learning) more so than sign-tracking behavior, and thus motivation itself. Prior studies have suggested major roles of ACh in motivation and behavioral flexibility separately^30,34–41^. Our results show that these receptors have opposing influences on the dynamics of motivation. Thus, the blockade of nAChRs augmented, while blockade of mAChRs reduced, the persistence of vigorous sign tracking behaviors (resulting in lever deflections) during omission. These effects resulted in near-significant differences in the number of successful trials during omission, in which nAChR blockade resulted in a trend of fewer rewarded (i.e., non-omitted) trials early in omission testing, and mAChR blockade trended towards overall increased rewarded trials. These opposing alterations were further characterized as alterations to flexible motivated responding, as changes to responses were not only seen both in the form of lever presses, but additionally within magazine entries and diverging differences in response microstructures in which each drug group was characterized by unique patterns of behaviors. Collectively, we find that ACh blockade in the NAc leads to a reorganized form of responding to CSs.

More specifically, nAChR blockade produced a bimodal effect over the course of testing. During initial days of omission, this manipulation resulted in more vigorous sign-tracking behaviors and lever deflections. This was also observed as a trend during overtraining, in which no omission schedule was imposed. mAChR blockade oppositely reduced such vigorous sign-tracking. Thus, one might conclude that ACh manipulations generally affected motivational attraction to the reward cues, resulting in a perseveration of sign-tracking responses that had been acquired over previous learning time with nAChR blockade and a converse reduction in perseverative sign-tracking with mAChR blockade.

However, with continued omission exposure and new learning, nAChR blockade caused a different effect. Animals with this manipulation began reducing their overall sign-tracking behaviors while increasing entries to the reward magazine during cue presentation. This effect was not observed during overtraining, nor was anything like this effect seen with mAChR blockade. This finding was surprising. We can conclude that there is an interaction between omission learning time and nAChR blockade, such that it encourages more vigorous sign-tracking initially but later encourages less sign-tracking and more goal-tracking-like behaviors. One broad possibly is that nAChR blockade led to a destabilization of the learned behavioral response, resulting first in greater engagement with the cue but later with less cue engagement. This destabilization effect cannot be a simple result of changes in learning rates, as those were not coherently affected, nor of changes in general motivation or reward valuation, as it was unique to late-phase omission. The fact that effects were bimodal—first enhancing and later reducing sign-tracking—suggests a form of greater flexibility in how motivation is behaviorally expressed under changing task conditions.

These findings are somewhat in line with recent results from Gheidi and colleagues suggesting that systemic mecamylamine infusion reduces sign-tracking responding^47^. However, unlike those experiments, we find that mecamylamine infusion in the NAc has a small but significant effect on non-sign-tracking behaviors. Furthermore, we did not observe any changes in sign-tracking or non-sign-tracking behaviors with scopolamine infusion as the mentioned study uncovered, which could indicate that behavioral alterations in sign-tracking responses are specifically due to ACh transmission in the NAc and do not capture the entirety of effects observed when these antagonists are given systemically.

Our results are also consistent with prior work implicating NAc ACh receptor activity in opposing roles in cue-motivation in a Pavlovian-to-Instrumental Transfer task (PIT). In a study by Collins and colleagues, a learned Pavlovian light cue and instrumental lever cue are presented as a compound cue. An opposing role of nAChRs and mAChRs were found, by which compound cue lever presses were increased or decreased, respectively, when these receptors were blocked, indicating differential modulation of motivation^39^. The “transfer” tested in PIT could be viewed as a type of flexibility on its own—this compound cue is new to the animal, and they must integrate known information into a new cue context. In PIT, it may be difficult to disentangle whether animals are less motivated, or if they are integrating information into their actions differently when ACh transmission changes. Sign-tracking affords us a different perspective of fundamental motivation itself. Thus, our results as well as those obtained in PIT may be a result of a potential cue-information integration and motivational flexibility mechanism of NAc ACh. This is not to say that ACh never contributes to the basic aspects of motivated behavior and incentive salience, as striatal ChIs have been shown to respond or pause firing during both cues and rewards^34,35,48–51^. Further, striatal ACh manipulations can alter motivated responding generally^37,40,41,52^, which we ourselves see during initial omission days and a bit during over-training. This role in response flexibility is similar to that seen in the striatum broadly^36,38,53–57^, and could be a key to understanding how ACh can manage behavioral adjustments through response modulation.

Based on these findings, we suggest that ACh transmission can serve as a balancing mechanism for how flexible animals are in how they exhibit attraction to reward cues, with the nAChRs biasing animals towards flexibility and, broadly speaking, the mAChRs biasing animals towards inflexibility. These opposing behavioral effects through the blockade of these receptors may not be consistent neurally, however. It is possible too that, rather than a general opposing mechanism, blockade of mAChRs as autoreceptors would increase the availability of ACh, while nAChR blockade could directly affect the efficacy of ACh signaling itself. Endogenous ACh release would affect this balance by acting on both receptors, but perhaps at different time courses or intra-striatal locations. In this line of reasoning, there may be orthogonal brain mechanisms for cue-driven motivation and for how malleable that motivation is in its behavioral expression. ACh may have a role in both processes, but there was a special reorganization of sign-tracking that occurred with nAChR blockade that cannot easily be attributed to general CS-related motivation alone. This manipulation changed the structure of the sign-tracking response throughout omission learning, and effects during late-phase omission experience—reduction in sign-tracking overall and an increase in goal-tracking—were only seen in this condition. Therefore, these receptors may relate to general cue-directed motivation, but they also relate to how that motivation is expressed behaviorally beyond just general motivation itself. An implication of this idea is that excessive or otherwise rigid motivational pursuit of goals could come about either through heightened motivational states or stable motivational states that lose amenability to change.

ACh receptor activity could alter the flexibility of motivation, including sign-tracking, through a few potential mechanisms. First, the effects of mecamylamine could have been the result of action at nAChRs on presynaptic dopaminergic terminals^27^, and mAChR regulation of nAChR activity^29^. ACh is known to be related to changes in phasic DA transmission^33,39^ which could ultimately reflect direct changes in DA-dependent sign-tracking responses^23^. Behaviorally relevant DA transmission is known to be regulated by nAChRs, dubbed as a “low-pass filter” of high-frequency dopaminergic stimulations^33^. These receptors have also been shown to regulate cue-evoked phasic DA in response to salient events such as reward-predictive cues^58^. The location of these receptors places them in a prime spot for direct modulation of known incentive salience and prediction error signaling, potential neural mechanisms underlying this type of responding. It is also known that ChIs pause their tonic firing during salient events, such as during rewards^34,35^. This pausing may allow for their tight regulation of DA to lift momentarily to allow for these types of signals^30^.

Omission is a special circumstance in which motivation under changing circumstances can be studied, as physical expressions of sign-tracking can change while incentive salience itself remains relatively intact. We view this careful balance as a form of flexibility within the sign-tracking response, as animals must change the microstructure of their responding while their motivations remain intact. Further work will be needed to resolve whether similar behavioral patterns are seen in other types of task changes that demand flexibility and response-inhibition. These could include extinction learning, reversal learning, or responding when negative prediction errors are introduced. Animals are known to adjust their sign-tracking behaviors under extinction and reversal conditions as assessed by lever-deflection readouts^59,60^, but it is unknown how the behavioral microstructure looks in these cases. Animals will also reduce sign-tracking when negative errors are introduced, such as in a condition where animals are yoked to the reward delivery schedule of a group exposed to the omission schedule and thus receive a similar distribution of reward and non-reward trials^12^. These conditions will be useful to determine how general the role of NAc ACh is in the changeability of sign-tracking, or how specific it is to the omission rule situation.

There are several studies that characterize sign-tracking to be habitual, generally inflexible, and insensitive to changes in outcome value^61–63^. In initial cases supporting this conclusion, devaluation of rewards occurred outside of the conditioning context, leading to potentially problematic integration of the devaluation learning and the task behavior. However, recent studies^8,9,64^ have found that when devaluation occurs within the testing chamber context, sign-tracking is quickly reduced and quite flexible. These studies, as well as some others^10^, lead us to conclude that sign-tracking is indeed sensitive to outcome values. The rapid sensitivity of animals to omission here and elsewhere^12^ further support this notion.

Concerning limitations, we observed that the pressing behaviors between ACSF groups in each experiment are not identical. We have found that sign-tracking can be variable across cohorts of animals, such that normal acquisition rates and asymptotic levels are difficult to predict cohort-to-cohort. Within experiments, we ran group cohorts simultaneously to help optimize group comparisons. Across experiments, however, we find it difficult to draw meaningful comparisons, and thus leave the apparent differences between ACSF groups unaccounted for. Further, we observed some differences in baseline sign-tracking levels ahead of drug infusion days. By normalizing per-rat to pre-infusion baseline levels, and through statistical inferences, we are confident that the effects of drug infusion are unlikely to be explained by preexisting sign-tracking (or magazine entry) tendencies. We also emphasize that that the behavioral microstructures resulting from infusion and omission are rather distinct between groups, making it unlikely that there were effects of the drugs that went beyond baseline behavioral patterns.

To summarize, NAc ACh is situated in a central position to support flexibility in dynamic environments, changing cue-reward relationships, and changing contexts. This brain mechanism both maintains and regulates motivation, but it can also be viewed as an entry point for understanding how to change the behavioral direction that motivation takes. Persistent maladaptive motivation responses and cue reactivity, such as those that occur in substance use disorders, could plausibly be targeted to advantage through the ACh system. Cue exposure therapies have promising results in treating those with substance use disorders^65–67^, but they often fail to produce substantial and lasting changes to real world cues in different contexts^68–71^. There is plenty of attention on ways to reduce cue-evoked reward craving and motivation in general. A different approach might be a method to change how that motivation is expressed behaviorally. If the ACh system can be targeted to cause a change in how cues are responded to, it might be beneficial as an aid to create the behavioral alteration that is needed to cease drug-seeking behaviors.

### Supplementary Information

Supplementary Information.

## Data Availability

The datasets used and/or analyzed in the conducted studies are available upon request from the corresponding author.
