# Supplementary material for: Nucleus accumbens core acetylcholine receptors modulate the balance of flexible and inflexible cue-directed motivation
Source: Sci Rep. 2023 Aug 17;13:13375. doi: 10.1038/s41598-023-40439-4 (PMC10435540; doi:10.1038/s41598-023-40439-4)
Supplement: Supplementary file 1 — Supplementary Information. [file 41598_2023_40439_MOESM1_ESM.docx]

**Nucleus accumbens core acetylcholine receptors modulate the balance of flexible and inflexible cue-directed motivation**

Erica S. Townsend*^1^, Kenneth A. Amaya^1,2^, Elizabeth B. Smedley^1,3^, Kyle S. Smith^1^

^1^ Dartmouth College, Department of Psychological and Brain Sciences, Hanover, NH, USA

^2^ Tufts University School of Medicine, Department of Neuroscience, Boston, MA, USA

^3^ Temple University, Department of Psychology, Philadelphia, PA, USA

*Corresponding Author:

Erica S. Townsend

erica.s.townsend.gr@dartmouth.edu

3 Maynard Street

Hanover, NH 03755

Phone: 603-646-1486

**Supplementary Materials:**

**Supplementary Figure 1:**


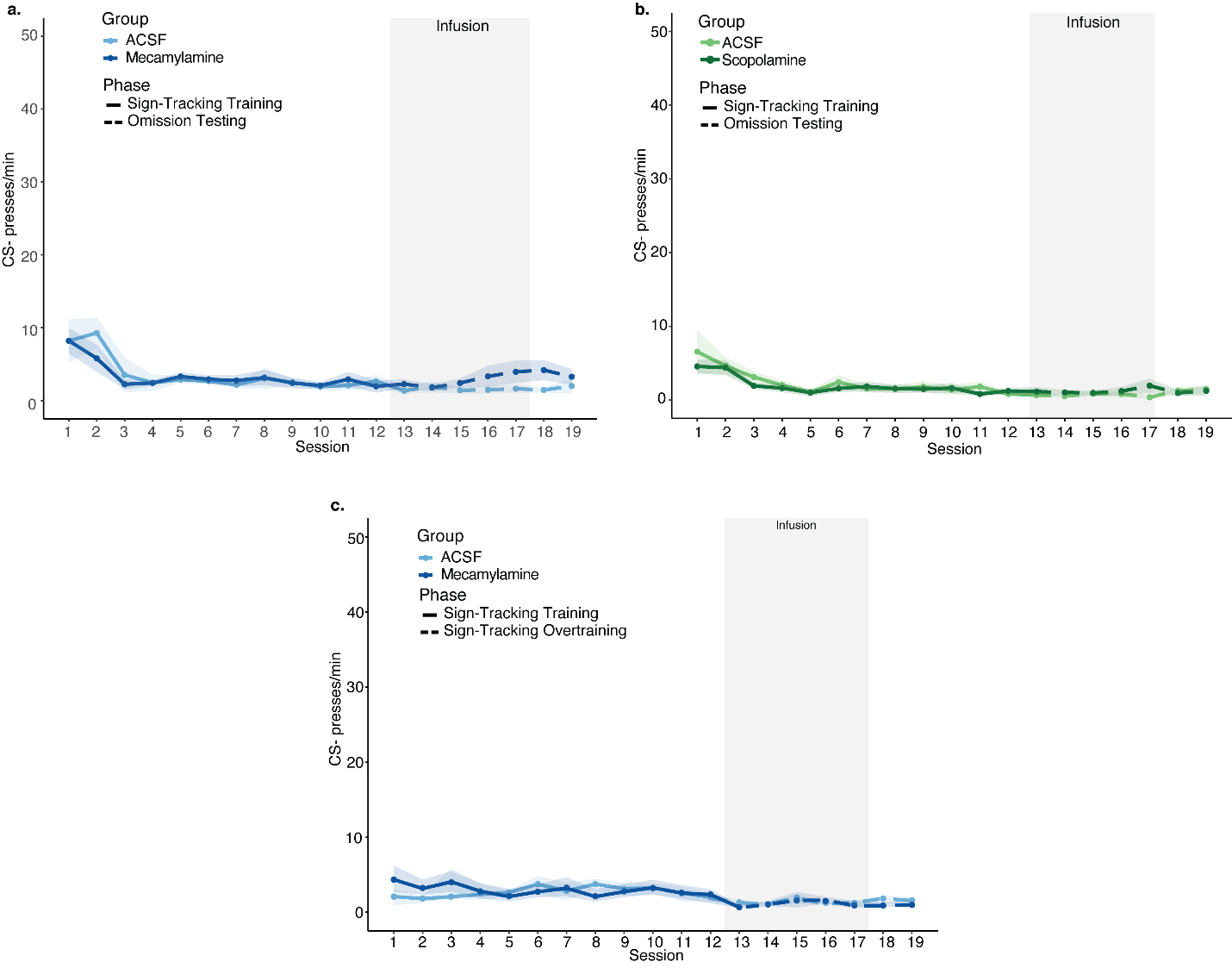


**Supplementary Figure 1:** CS- lever presses per minute (PPM) for Experiments 1, 2, and 3. (A) PPM on the CS- lever in Experiment 1 over the 12 sign-tracking training sessions (sessions 1-12, solid line) and 7 omission testing sessions (sessions 13-19, dotted line) for the mecamylamine (dark blue) and ACSF (light blue) groups. Shaded sessions indicate infusion sessions. (B) PPM on the CS- lever in Experiment 2 over the 12 sign-tracking training sessions (sessions 1-12, solid line) and 7 omission testing sessions (sessions 13-19, dotted line) for the scopolamine (dark green) and ACSF (light green) groups. Shaded sessions indicate infusion sessions. (C) PPM on the CS- lever in Experiment 3 over the 12 sign-tracking training sessions (sessions 1-12) and 7 omission testing sessions (sessions 13-19) for the mecamylamine (dark blue) and ACSF (light blue) groups. Shaded sessions indicate infusion sessions. For all graphs, lines show mean and error shows ±SEM.

**Supplementary Figure 2:**

**
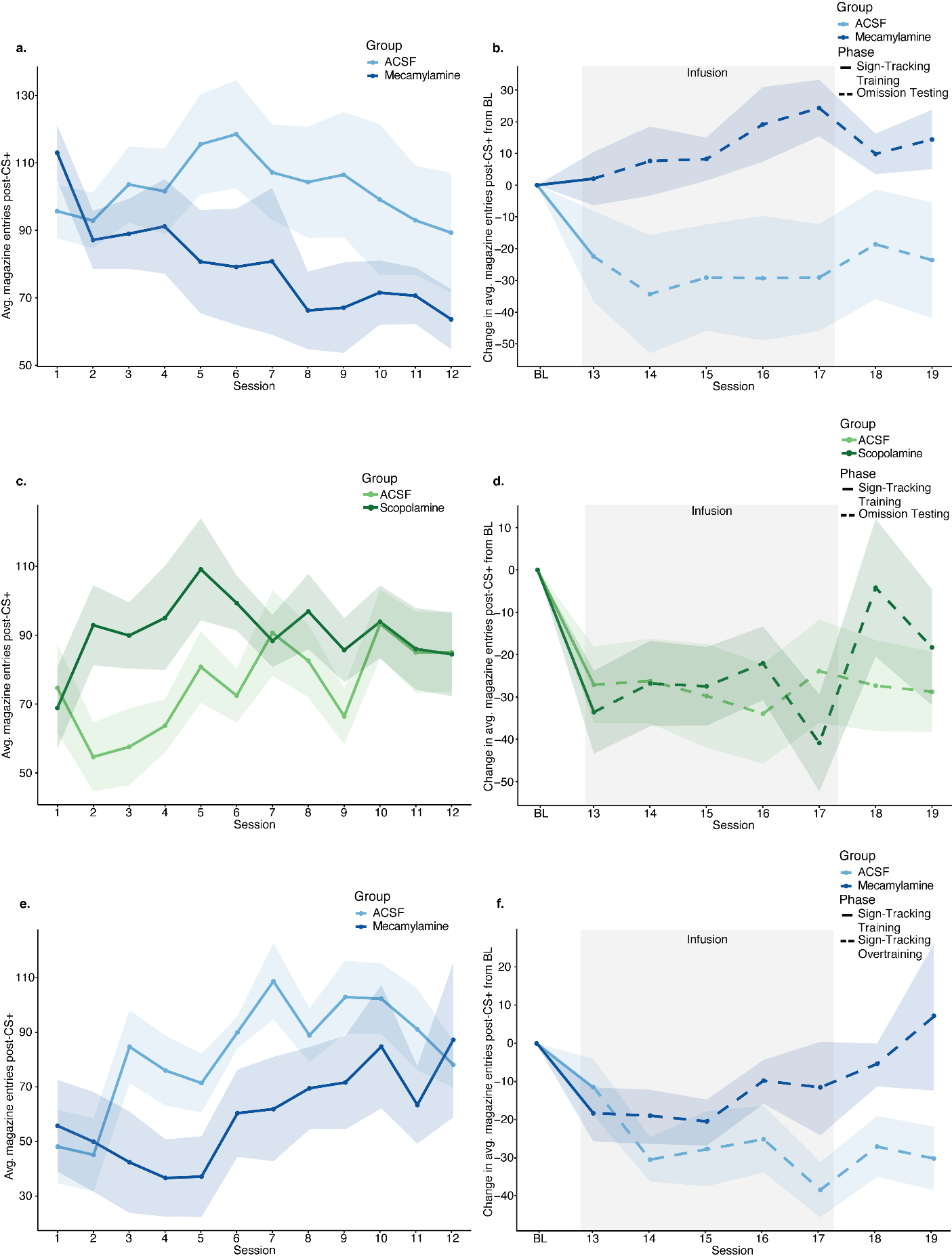
**

**Supplementary Figure 2:** Magazine entries during the 10 second period post-CS+ presentation, or pellet delivery period. (A) Experiment 1 average magazine entries during the 10 second post-CS+ presentation period over the 12 sign-tracking acquisition training sessions for mecamylamine (dark blue) and ACSF (light blue) groups. (B) Experiment 1 change in average magazine entries during the 10 second post-CS+ period from a 3-session baseline created for each animal by averaging magazine entries on the last 3 sessions of sign-tracking acquisition. All 7 omission sessions were normalized to the 3-session baseline for the mecamylamine (dark blue) and ACSF (light blue) groups. Gray shaded sessions indicate infusion sessions. (C) Experiment 2 average magazine entries during the 10 second post-CS+ presentation period over the 12 sign-tracking acquisition training sessions for scopolamine (dark green) and ACSF (light green) groups. (D) Experiment 2 change in average magazine entries during the 10 second post-CS+ period from a 3-session baseline created for each animal by averaging magazine entries on the last 3 sessions of sign-tracking acquisition. All 7 omission sessions were normalized to the 3-session baseline for the scopolamine (dark green) and ACSF (light green) groups. Gray shaded sessions indicate infusion sessions. (D) Experiment 3 average magazine entries during the 10 second post-CS+ presentation period over the 12 sign-tracking acquisition training sessions for mecamylamine (dark blue) and ACSF (light ble) groups. (E) Experiment 3 change in average magazine entries during the 10 second post-CS+ period from a 3-session baseline created for each animal by averaging magazine entries on the last 3 sessions of sign-tracking acquisition. All 7 sign-tracking overtraining sessions were normalized to the 3-session baseline for the mecamylamine (dark blue) and ACSF (light blue) groups. Gray shaded sessions indicate infusion sessions. For all graphs, points show mean of each session and error ribbons show ±SEM.

**Supplementary Table 1:**

| **Sign-Tracking Training (Sessions 1-12)** | | | |
| --- | --- | --- | --- |
| **Predictor** | **Estimate** | **CI** | **P-Value** |
| Group | -0.14 | -0.79 – 0.52 | 0.682 |
| Session | -1.41 | -1.90 – -0.92 | **<0.001** |
| Group × Session | 0.24 | -0.24 – 0.73 | 0.326 |
| **Omission Testing (Baseline + Normalized Sessions 13-19)** | | | |
| **Predictor** | **Estimate** | **CI** | **P-Value** |
| Group | 0.63 | -0.26 – 1.52 | 0.164 |
| Session | 0.30 | -0.27 – 0.87 | 0.304 |
| Group × Session | 0.35 | -0.22 – 0.92 | 0.231 |

**Supplementary Table 1:** Linear mixed model estimates, confidence intervals (CI), and p-values for group, session, and group and session interaction predictors for the dependent value of CS- lever presses per minute in Experiment 1. Models were created for two phases in the experiment: sign-tracking training (days 1-12) and change from a 3-day training baseline (average of PPM in the last 3 days of sign-tracking training) + omission testing (days 13-19). Significant values are indicated in bold.

**Supplementary Table 2:**

| **Sign-Tracking Training (Sessions 1-12)** | | | |
| --- | --- | --- | --- |
| **Predictor** | **Estimate** | **CI** | **P-Value** |
| Group | -11.13 | -23.90 – 1.65 | 0.088 |
| Session | -6.63 | -13.99 – 0.72 | 0.077 |
| Group × Session | -5.17 | -12.53 – 2.18 | 0.167 |
| **Omission Testing (Baseline + Normalized Sessions 13-19)** | | | |
| **Predictor** | **Estimate** | **CI** | **P-Value** |
| Group | 17.01 | 2.40 – 31.62 | **0.023** |
| Session | 0.96 | -3.92 – 5.83 | 0.699 |
| Group × Session | 4.54 | -0.33 – 9.41 | 0.067 |

**Supplementary Table 2:** Linear mixed model estimates, confidence intervals (CI), and p-values for group, session, and group and session interaction predictors for the dependent value of average magazine entries during the 10 second post-CS+ period (reward delivery period) in Experiment 1. Models were created for two phases in the experiment: sign-tracking training (days 1-12), and change from a 3-day training baseline (average of magazine entries in the last 3 days of sign-tracking training) during omission testing. Significant values are indicated in bold.

**Supplementary Table 3:**

| **Sign-Tracking Training (Sessions 1-12)** | | | |
| --- | --- | --- | --- |
| **Predictor** | **Estimate** | **CI** | **P-Value** |
| Group | -0.21 | -0.93 – 0.50 | 0.559 |
| Session | -1.03 | -1.53 – -0.54 | **<0.001** |
| Group × Session | 0.19 | -0.30 – 0.69 | 0.449 |
| **Omission Testing (Baseline + Normalized Sessions 13-19)** | | | |
| **Predictor** | **Estimate** | **CI** | **P-Value** |
| Group | 0.15 | -0.25 – 0.56 | 0.463 |
| Session | 0.07 | -0.14 – 0.29 | 0.499 |
| Group × Session | -0.01 | -0.23 – 0.20 | 0.899 |

**Supplementary Table 3:** Linear mixed model estimates, confidence intervals (CI), and p-values for group, session, and group and session interaction predictors for the dependent value of CS- lever presses per minute in Experiment 2. Models were created for two phases in the experiment: sign-tracking training (days 1-12) and change from a 3-day training baseline (average of PPM in the last 3 days of sign-tracking training) + omission testing (days 13-19). Significant values are indicated in bold.

**Supplementary Table 4:**

| **Sign-Tracking Training (Sessions 1-12)** | | | |
| --- | --- | --- | --- |
| **Predictor** | **Estimate** | **CI** | **P-Value** |
| Group | 15.31 | -3.27 – 33.88 | 0.106 |
| Session | 8.15 | 1.39 – 14.91 | **0.018** |
| Group × Session | -7.63 | -17.19 – 1.92 | 0.117 |
| **Omission Testing (Baseline + Normalized Sessions 13-19)** | | | |
| **Predictor** | **Estimate** | **CI** | **P-Value** |
| Group | 2.98 | -16.07 – 22.04 | 0.757 |
| Session | -5.48 | -12.41 – 1.44 | 0.120 |
| Group × Session | 4.99 | -4.80 – 14.78 | 0.315 |

**Supplementary Table 4:** Linear mixed model estimates, confidence intervals (CI), and p-values for group, session, and group and session interaction predictors for the dependent value of average magazine entries during the 10 second post-CS+ period (reward delivery period) in Experiment 2. Models were created for two phases in the experiment: sign-tracking training (days 1-12), and change from a 3-day training baseline (average of magazine entries in the last 3 days of sign-tracking training) during omission testing. Significant values are indicated in bold.

**Supplementary Table 5:**

| **Sign-Tracking Training (Sessions 1-12)** | | | |
| --- | --- | --- | --- |
| **Predictor** | **Estimate** | **CI** | **P-Value** |
| Group | 0.03 | -0.70 – 0.76 | 0.939 |
| Session | -0.19 | -0.68 – 0.30 | 0.440 |
| Group × Session | -0.25 | -0.73 – 0.24 | 0.319 |
| **Sign-Tracking Overtraining (Baseline + Normalized Sessions 13-19)** | | | |
| **Predictor** | **Estimate** | **CI** | **P-Value** |
| Group | -0.16 | -0.61 – 0.29 | 0.478 |
| Session | -0.21 | -0.45 – 0.03 | 0.085 |
| Group × Session | -0.08 | -0.32 – 0.16 | 0.510 |

**Supplementary Table 5:** Linear mixed model estimates, confidence intervals (CI), and p-values for group, session, and group and session interaction predictors for the dependent value of CS- lever presses per minute in Experiment 3. Models were created for two phases in the experiment: sign-tracking training (days 1-12) and change from a 3-day training baseline (average of PPM in the last 3 days of sign-tracking training) + sign-tracking overtraining (days 13-19). Significant values are indicated in bold.

**Supplementary Table 6:**

| **Sign-Tracking Training (Sessions 1-12)** | | | |
| --- | --- | --- | --- |
| **Predictor** | **Estimate** | **CI** | **P-Value** |
| Group | -11.12 | -24.48 – 2.25 | 0.103 |
| Session | 12.77 | 3.04 – 22.50 | **0.010** |
| Group × Session | -0.21 | -9.94 – 9.52 | 0.966 |
| **Omission Testing (Baseline + Normalized Sessions 13-19)** | | | |
| **Predictor** | **Estimate** | **CI** | **P-Value** |
| Group | 7.51 | -5.25 – 20.26 | 0.248 |
| Session | 0.57 | -6.78 – 5.63 | 0.856 |
| Group × Session | 6.20 | -0.01 – 12.40 | 0.065 |

**Supplementary Table 6:** Linear mixed model estimates, confidence intervals (CI), and p-values for group, session, and group and session interaction predictors for the dependent value of average magazine entries during the 10 second post-CS+ period (reward delivery period) in Experiment 3. Models were created for two phases in the experiment: sign-tracking training (days 1-12), and change from a 3-day training baseline (average of magazine entries in the last 3 days of sign-tracking training) during sign-tracking overtraining. Significant values are indicated in bold.

**Supplementary Table 7:**

| **Lever Bites** | | | |
| --- | --- | --- | --- |
| **Predictor** | **Estimate** | **CI** | **P-Value** |
| Group | 1.22 | -1.05 – 3.48 | 0.288 |
| Session | -3.00 | -3.95 – -2.06 | **< 0.001** |
| Group × Session | -0.15 | -1.09 – 0.80 | 0.756 |
| **Lever** **Grabs** | | | |
| **Predictor** | **Estimate** | **CI** | **P-Value** |
| Group | -0.92 | -2.34 – 0.51 | 0.203 |
| Session | -2.15 | -3.18 – -1.11 | **< 0.001** |
| Group × Session | -0.09 | -3.18 – -1.11 | 0.859 |
| **Lever Contacts** | | | |
| **Predictor** | **Estimate** | **CI** | **P-Value** |
| Group | -0.97 | -1.81 – -0.13 | **0.024** |
| Session | 1.82 | 1.01 – 2.63 | **< 0.001** |
| Group × Session | -0.28 | -1.09 – 0.53 | 0.496 |
| **Lever Sniffs** | | | |
| **Predictor** | **Estimate** | **CI** | **P-Value** |
| Group | -0.45 | -1.70 – 0.80 | 0.477 |
| Session | 2.14 | 1.38 – 2.90 | **< 0.001** |
| Group × Session | -0.05 | -0.81 – 0.71 | 0.894 |
| **Lever Orients** | | | |
| **Predictor** | **Estimate** | **CI** | **P-Value** |
| Group | -0.17 | -0.66 – 0.32 | 0.486 |
| Session | 0.17 | -0.08 – 0.42 | 0.177 |
| Group × Session | -0.18 | -0.43 – 0.07 | 0.151 |
| **Magazine-Directed Behaviors** | | | |
| **Predictor** | **Estimate** | **CI** | **P-Value** |
| Group | 1.43 | 0.36 – 2.49 | **0.010** |
| Session | 0.93 | 0.28 – 1.59 | **0.006** |
| Group × Session | 0.82 | 0.16 – 1.48 | **0.015** |
| **Non-CS+ Directed Behaviors** | | | |
| **Predictor** | **Estimate** | **CI** | **P-Value** |
| Group | -0.13 | -0.37 – 0.12 | 0.302 |
| Session | 0.08 | -0.15 – 0.32 | 0.480 |
| Group × Session | -0.07 | -0.31 – 0.16 | 0.541 |

**Supplementary Table 7:** Linear mixed model estimates, confidence intervals (CI), and p-values for group, session, and group and session interaction predictors for the 7 behaviors scored in Experiment 1. Models analyzed the effects of dependent variable responding (scored behavior categories) by fixed effects of experimental group and session while accounting for individual starting values. Significant values are indicated in bold.

**Supplementary Table 8:**

| **Lever Bites** | | | |
| --- | --- | --- | --- |
| **Predictor** | **Estimate** | **CI** | **P-Value** |
| Group | -1.35 | -2.64 – -0.05 | **0.042** |
| Session | -1.38 | -2.24 – -0.52 | **0.002** |
| Group × Session | -0.17 | -1.03 – 0.69 | 0.697 |
| **Lever** **Grabs** | | | |
| **Predictor** | **Estimate** | **CI** | **P-Value** |
| Group | -1.64 | -2.86 – -0.42 | **0.009** |
| Session | -1.35 | -1.89 – -0.81 | **< 0.001** |
| Group × Session | -0.18 | -0.72 – 0.37 | 0.522 |
| **Lever Contacts** | | | |
| **Predictor** | **Estimate** | **CI** | **P-Value** |
| Group | 0.21 | -0.60 – 1.01 | 0.608 |
| Session | 0.66 | 0.12 – 1.20 | **0.018** |
| Group × Session | 0.31 | -0.23 – 0.85 | 0.262 |
| **Lever Sniffs** | | | |
| **Predictor** | **Estimate** | **CI** | **P-Value** |
| Group | 1.15 | 0.08 – 2.22 | **0.035** |
| Session | 0.94 | 0.39 – 1.50 | **0.001** |
| Group × Session | 0.06 | -0.50 – 0.61 | 0.840 |
| **Lever Orients** | | | |
| **Predictor** | **Estimate** | **CI** | **P-Value** |
| Group | 0.51 | 0.05 – 0.97 | **0.029** |
| Session | 0.24 | -0.07 – 0.55 | 0.121 |
| Group × Session | 0.02 | -0.29 – 0.33 | 0.904 |
| **Magazine-Directed Behaviors** | | | |
| **Predictor** | **Estimate** | **CI** | **P-Value** |
| Group | 0.57 | -0.94 – 2.08 | 0.454 |
| Session | 0.86 | 0.34 – 1.38 | **0.002** |
| Group × Session | -0.11 | -0.63 – 0.41 | 0.684 |
| **Non-CS+ Directed Behaviors** | | | |
| **Predictor** | **Estimate** | **CI** | **P-Value** |
| Group | 0.49 | -0.21 – 1.18 | 0.165 |
| Session | 0.07 | -0.52 – 0.66 | 0.816 |
| Group × Session | 0.13 | -0.46 – 0.72 | 0.658 |

**Supplementary Table 8:** Linear mixed model estimates, confidence intervals (CI), and p-values for group, session, and group and session interaction predictors for the 7 behaviors scored in Experiment 2. Models analyzed the effects of dependent variable responding (scored behavior categories) by fixed effects of experimental group and session while accounting for individual starting values. Significant values are indicated in bold.
